# Neurocognitive and Autonomic Signatures of Performance Under Motivational Stress: An Integrated Psychophysiological Analysis of Reward and Punishment in Shooting Performance

**DOI:** 10.1111/sms.70227

**Published:** 2026-02-16

**Authors:** Ming‐Yang Cheng, Kuo‐Pin Wang, Tsung‐Min Hung, Calvin Lu, Hyuk Oh, Ying Ying Tan, Bradley Hatfield

**Affiliations:** ^1^ Department of Sports Science Research Taiwan Institute of Sports Science Kaohsiung Taiwan; ^2^ Master Program of Sport Facility and Health Promotion National Taiwan University Taipei Taiwan; ^3^ Department of Physical Education and Sport Sciences National Taiwan Normal University Taipei Taiwan; ^4^ Institute for Research Excellence in Learning Science National Taiwan Normal University Taipei Taiwan; ^5^ Department of Kinesiology University of Maryland at College Park College Park Maryland USA; ^6^ Neuroscience and Cognitive Sciences Program University of Maryland at College Park College Park Maryland USA

**Keywords:** EEG, heart rate variability, motivation, neurofeedback, performance under pressure, stress and cognitive performance

## Abstract

Motivational framing—such as reward and punishment—critically shapes performance under pressure, yet the underlying neurocognitive and autonomic mechanisms remain unclear. Guided by the cognitive–affective–motor (CAM) model and psychomotor efficiency theory (PET), this study examined how motivational context modulates brain–body dynamics during high‐pressure precision performance. Using a within‐subject design, elite marksmen performed a simulated shooting task under reward, punishment, and neutral conditions. Neurophysiological markers were assessed across four domains: affective regulation (frontal alpha asymmetry [FAA], eyeblink startle [EBS]), cognitive control (feedback‐related negativity [fERN], frontal midline theta), motor readiness (sensorimotor rhythm [SMR], fronto‐temporal coherence), and autonomic flexibility (heart rate variability [HRV]). Reward framing elicited a coordinated brain–body state marked by elevated SMR and HRV, greater left‐frontal activation, and reduced fERN and coherence—supporting focus, emotional control, and movement stability. Punishment elicited defensive arousal, heightened error sensitivity, and disrupted cortical communication, particularly in lower performers. These results demonstrate that motivational incentives recalibrate neurocognitive and autonomic systems, shaping performance resilience or vulnerability. The identified markers represent viable targets for neurofeedback and biofeedback interventions aimed at enhancing resilience, attentional control, and execution in elite sport performance.

## 1 Introduction

Optimal performance under high‐pressure conditions—whether in elite sport, military operations, or other precision‐demanding tasks—requires more than technical skill. Success depends on the coordinated regulation of emotional, cognitive, motor, and autonomic systems that sustain stability under stress [1]. Among the factors shaping performance in such contexts, motivational contingencies such as reward and punishment are especially influential. These contingencies simulate real‐world stakes and alter psychological states [2].

Reward and punishment engage distinct neurobiological pathways. Reward framing typically activates dopaminergic circuits that promote approach motivation, focused attention, and goal‐directed behavior. In contrast, punishment framing heightens arousal and defensive control processes, often involving the amygdala and anterior cingulate cortex [3]. These mechanisms suggest that motivational context can recalibrate how performers regulate attention, emotion, and action under pressure.

Importantly, motivational effects extend beyond emotional responses. They drive dynamic adjustments across prefrontal regulatory networks, autonomic function, and sensorimotor systems—mechanisms essential for sustaining efficiency under stress. For example, performance‐contingent rewards enhance cognitive control and efficiency [4], whereas reward cues improve attentional focus and motor readiness even outside conscious awareness [5]. Anticipating a reward can reduce anxiety, enabling prefrontal networks to sustain focus, autonomic regulation to maintain physiological calm, and sensorimotor systems to execute movements with greater consistency [6]. Nevertheless, the specific neurocognitive and autonomic mechanisms through which motivational incentives recalibrate brain–body coordination remain incompletely understood.

Theoretical models provide a framework for interpreting these dynamics. The cognitive–affective–motor (CAM) model [7] proposes that optimal performance emerges from dynamic cross‐domain regulation linking emotional regulation, executive control, and motor efficiency. The psychomotor efficiency theory (PET) complements this view by emphasizing neural economy: experts under pressure exhibit reduced cortical interference and refined sensorimotor synchronization [8]. Together, CAM and PET predict that motivational framing influences performance not by acting on a single system, but by coordinating affective, cognitive, motor, and autonomic processes.

Electroencephalography (EEG) has been widely used to examine these processes. Sensorimotor rhythm (SMR) reflects motor readiness and attentional stability, with higher SMR power linked to reduced neuromuscular noise and more consistent motor output [9, 10]. Frontal midline (Fz) theta, associated with executive functioning and conflict monitoring, increases under evaluative or high‐demand conditions, reflecting sustained cognitive control [11, 12]. Alpha power (8–13 Hz) at the left temporal site (T3) reflects verbal–analytic activity. In skilled motor tasks, higher T3 alpha indicates reduced verbal control and greater neural efficiency, whereas lower T3 alpha reflects intrusive self‐talk and diminished automaticity [13, 14, 15].

Other EEG markers capture affective and cognitive regulation. Fronto‐temporal coherence, particularly alpha‐band connectivity between Fz and T3, reflects the coupling of executive control and verbal–analytic processes. Excessive coherence has been linked to verbal intrusion and over‐control, which can disrupt motor automaticity [16, 17]. Similarly, T3–Pz coherence indexes the balance between verbal–analytic processing and visuomotor integration, with reduced coherence reflecting more automatic and efficient movement control [13, 14]. Frontal alpha asymmetry (FAA) provides a complementary index of motivational orientation: greater left‐frontal activation is associated with approach motivation and resilience, whereas right‐frontal dominance is linked to avoidance and anxiety [18]. Collectively, these indices provide a multidimensional framework for assessing attentional regulation, affective stability, and motor efficiency.

Despite these advances, prior research has often relied on isolated measures or categorical expert–novice comparisons (e.g., Refs. [19, 20]). Such approaches obscure the dynamic interplay of neural markers within homogeneous expert groups. This limitation highlights the need for refined methods that examine integrated, context‐dependent modulation of neurocognitive and psychophysiological processes in elite performers [21].

**FIGURE 1:**
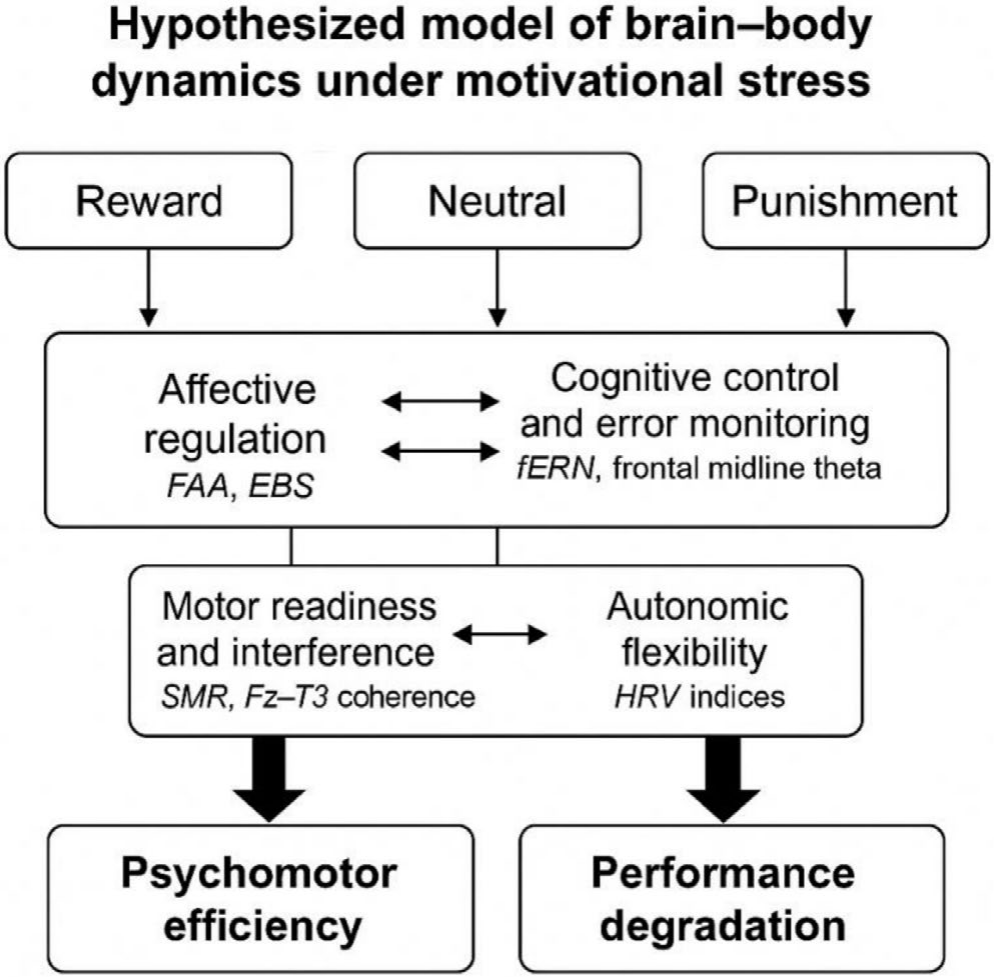
This model delineates how motivational contexts (reward, neutral, punishment) modulate four interdependent systems underpinning elite performance: Affective regulation (frontal alpha asymmetry [FAA], eyeblink startle [EBS]), cognitive control and error monitoring (feedback‐related negativity [fERN], frontal midline theta), motor readiness and interference (sensorimotor rhythm [SMR], Fz–T3 alpha coherence), and autonomic flexibility (heart rate variability [HRV] indices, LF/HF ratio, RMSSD, SDNN). Reward conditions are hypothesized to align these systems, promoting psychomotor efficiency, whereas punishment disrupts their coordination, precipitating performance degradation.

Anchored in the CAM model, the present study addresses this gap by investigating how motivational contingencies—reward, punishment, and neutral conditions—dynamically shape cognitive control, affective regulation, motor efficiency, and autonomic flexibility in a uniform cohort of highly trained marksmen. Unlike prior work focusing on single indices, we adopt a psychophysiological framework that integrates multiple measures: feedback‐related negativity (fERN) for cognitive control, eyeblink startle response (EBS) for affective arousal, FAA and frontal midline theta for affective and cognitive regulation, SMR and fronto‐temporal coherence for motor readiness, and heart rate variability (HRV) for autonomic function. Using a within‐subject design, we hypothesize that reward contexts will enhance psychomotor efficiency—marked by increased FAA and SMR, reduced fERN and coherence, and elevated HRV—while punishment will disrupt this balance. By integrating neurocognitive and autonomic indices within a unified psychophysiological framework, this study advances understanding of the interconnected mechanisms that distinguish performance resilience from vulnerability under motivational stress (Figure 1).

## 2 Methods

### 2.1 Participants

Thirty‐five right‐handed male participants (mean age = 21.8 ± 4.5 years), all affiliated with the Reserve Officers' Training Corps (ROTC) at the University of Maryland, participated in this study. In International Practical Shooting Confederation (IPSC) classification, Class B corresponds to shooters achieving 60%–74.9% of the Grand Master hit‐factor standard, denoting nationally competitive proficiency. Within the ROTC training framework, these criteria meet operational definitions of “elite” performers [22]. Handedness was confirmed using the Edinburgh Handedness Inventory (EHI) [23]. All participants were certified as Class B shooters under IPSC standards, with at least 2 years of pistol shooting experience (*M* = 3.67 ± 2.82 years). Written informed consent was obtained in accordance with the University of Maryland Institutional Review Board (IRB). An a priori power analysis was conducted using G*Power 3.1 [24] for a repeated‐measures ANOVA with three within‐subject conditions (reward, punishment, neutral). Based on prior work reporting a large effect size for SMR power in elite performers [25], we adopted an effect size of *f* = 0.675 (partial *η* ^2^ ≈ 0.31), with *α* = 0.05 and desired power set at 0.80. The analysis indicated that approximately 12 participants would be sufficient to detect a significant within‐subject effect. With 35 participants, the achieved power exceeded 0.99, ensuring robust sensitivity to detect condition‐related differences in neural and behavioral markers. Participants were classified as superior or inferior performers using a median split on their total monetary balance at the end of the experiment. This balance, reflecting accumulated gains and losses across all conditions, served as an index of overall task success and was used as the grouping factor in subsequent 3 × 2 repeated‐measures ANOVAs (Condition × Performance Group).

### 2.2 Shooting Task

**FIGURE 2:**
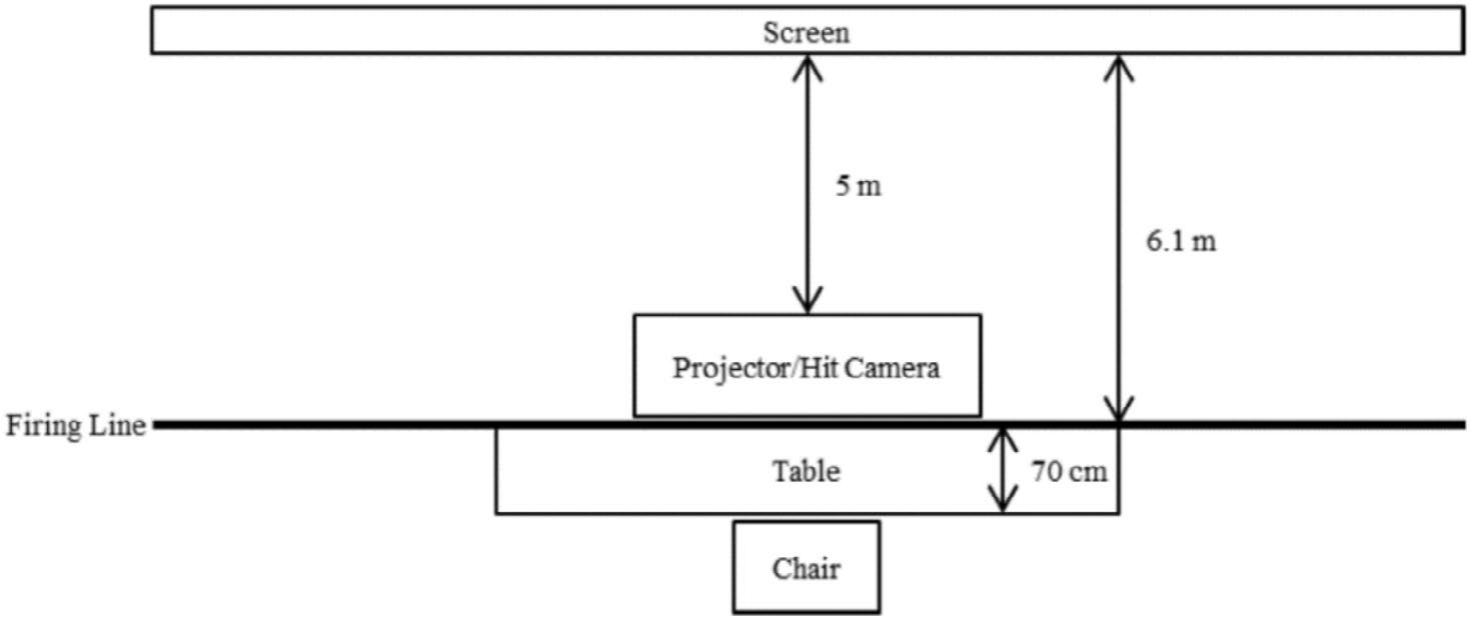
Experimental room set‐up.

**FIGURE 3:**
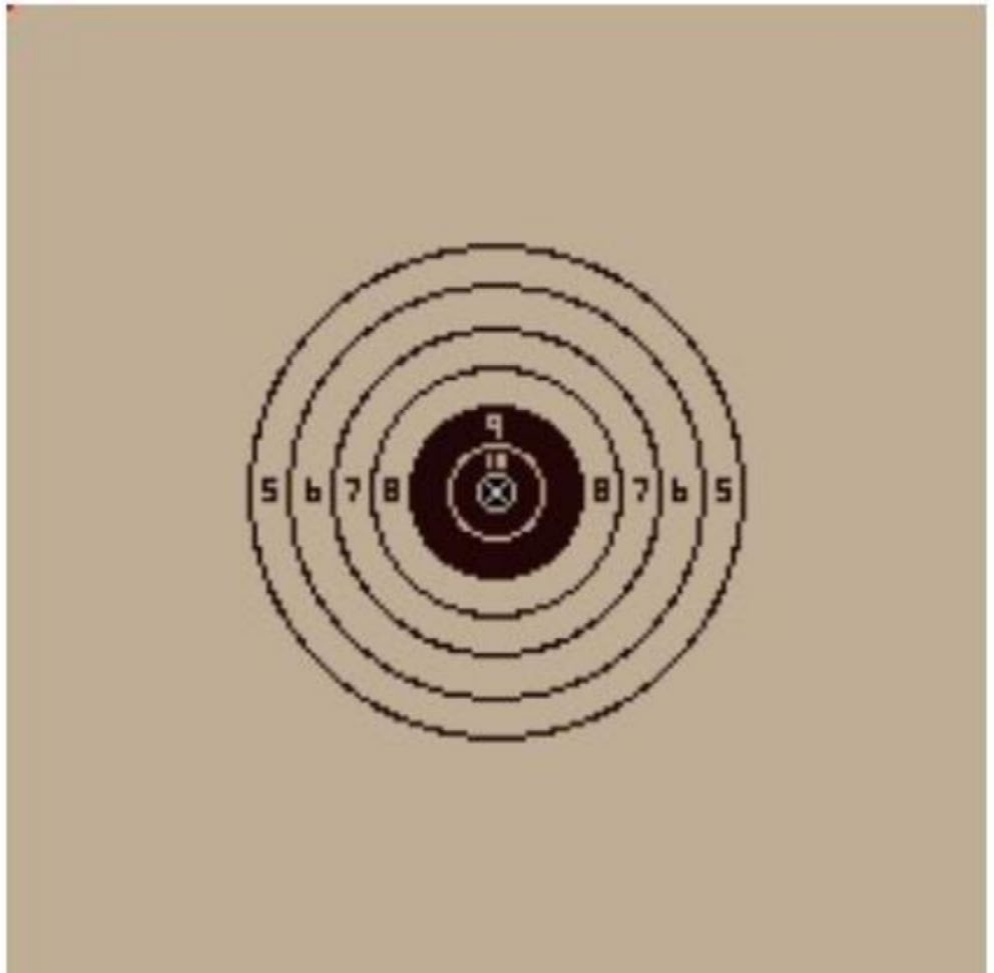
Image of presented bullseye target. The shaded area measured 8 cm in diameter when projected onto the screen.

The shooting task was conducted using the Meggitt Firearms Training System (FATS), a validated simulation platform providing real‐time shot detection and audiovisual feedback. To standardize conditions, the training pistol was configured without recoil, and participants manually racked the slide after each shot. Participants were seated 6.1 m from the projection screen, consistent with FATS simulation standards, ensuring ecological validity while maintaining laboratory control (Figure 2). At this distance, the centrally projected bullseye target measured 8 cm in diameter (~1.5° visual angle) on a 2.4 m × 1.8 m display. The target comprised 10 concentric scoring rings (0.8 cm width each), numbered from 10 (center) to 1 (outermost), rendered in alternating black–white contrast bands with the central 10‐ring highlighted in red to enhance visibility and feedback salience (Figure 3).

Each trial began with a fixation cross (“+”) presented for 500 ms at the target center to standardize gaze direction. The bullseye then remained visible for 4 s as participants aimed, with the trigger pull marking trial termination. Performance feedback (“HIT,” “MISS,” “+ $1,” “– $1,” or “$0”) appeared 1 s after shot execution for 1 s before the next trial commenced. This timing ensured that EEG epochs captured four consecutive 1‐s aiming segments prior to the shot and one 1‐s post‐shot segment, aligning with the neurophysiological analysis windows described in Section 2.7 Data Analysis.

Behavioral performance was assessed using outcome‐based indices (total shooting score, performance consistency, motor control precision) and process‐oriented measures (accuracy, precision, gunpoint jerkiness). Total score reflected cumulative points across 30 shots per condition, consistency was indexed by the standard deviation of shot scores, and motor control precision captured temporal stability across consecutive shots. Accuracy reflected proximity to the bullseye, precision was defined as radial error (dispersion around the shot centroid), and gunpoint jerkiness indexed pre‐shot aimpoint variability. Together, these measures provided complementary indices of task success and underlying motor control quality.

### 2.3 Psychophysiological Assessments

#### 2.3.1 Subjective Assessments

State anxiety and perceived workload were assessed using validated paper‐and‐pencil instruments administered immediately after each task block to capture in‐session psychological states. The State‐Trait Anxiety Inventory‐State Form (STAI‐S) [26] was used to measure transient anxiety and tension “at the moment.” This instrument has demonstrated excellent internal consistency (*α* ≈ 0.90) and sensitivity to stress manipulations in performance contexts, making it well‐suited for evaluating condition‐specific fluctuations in anxiety across reward, punishment, and neutral conditions.

Subjective workload was evaluated using the National Aeronautics and Space Administration Task Load Index (NASA‐TLX) [27], which assesses six dimensions of workload: mental demand, physical demand, temporal demand, effort, performance, and frustration. The NASA‐TLX has shown high reliability and convergent validity across both laboratory and operational motor‐control tasks. Paper‐based administration was selected to minimize interference with the shooting interface and preserve participants' focus, whereas still providing immediate post‐block ratings.

To validate the motivational manipulation, a post‐experiment check was conducted in which participants reflected on whether they perceived and responded differently across conditions and ranked the reward, punishment, and neutral conditions in terms of perceived stress and enjoyment.

#### 2.3.2 Objective Assessment

##### 2.3.2.1 EEG Recording

EEG data were recorded using a BrainVision amplifier system (Brain Products GmbH, Germany) following the protocol outlined by Cheng et al. [25]. EEG signals were continuously acquired from 32 scalp electrodes positioned according to the international 10–20 system [28], ensuring standardized spatial coverage of cortical activity. Signals were initially referenced to the left earlobe (A1) and re‐referenced offline to the average of A1 and A2. Data were sampled at 500 Hz and filtered using a 0.1–40 Hz bandpass filter and a 60 Hz notch filter to attenuate line noise.

Vertical and horizontal electrooculographic (VEOG and HEOG) activity was recorded using four additional electrodes placed around the eyes to support ocular artifact correction. Electrode impedances were kept below 5 kΩ throughout the session to ensure signal quality.

Prior to the shooting task, a 4‐s pre‐shot window was used to capture aimpoint stability, which served as an index of pre‐movement motor control. Baseline EEG activity was then recorded under resting conditions: participants were instructed to sit still for 2 min with their eyes open, followed by 2 min with their eyes closed. Upon completion of baseline recording, the experimental task commenced.

##### 2.3.2.2 Startle Reflex—Electromyography (EMG) Assessment

Eyeblink startle (EBS) amplitude, measured within the 0–1000 ms window following the trigger pull, indexed affective and neurophysiological reactivity across motivational contexts. The reflex was automatically elicited by the 120 dB, 50 ms gunshot sound produced by the simulator at the exact moment of trigger pull; this acoustic event served as the startle probe and was simultaneously recorded into the EEG system for precise temporal alignment. Surface EMG was collected from the right orbicularis oculi, with electrodes positioned below the inner and outer canthi of the right eye. Startle amplitude was defined as the peak‐to‐peak EMG voltage within the reflex window, following established startle‐response protocols [29].

##### 2.3.2.3 Heart Rate Variability–Electrocardiography (ECG) Assessment

HRV analyses were conducted using a hybrid pipeline in which QRSTool v1.2.2 (Allen, University of Arizona) was used exclusively for R‐peak detection and visual editing, whereas all subsequent HRV processing and analysis were performed in MATLAB R2022a (MathWorks Inc., Natick, MA). This distinction ensures that QRSTool was not used for HRV computation itself, but only for accurate beat detection and artifact correction.

Artifact correction followed Task Force guidelines [30]: ectopic or missing beats were identified visually and corrected by cubic spline interpolation when inter‐beat intervals (IBI) deviated by > 20% from the local mean. To minimize potential bias from manual inspection, all corrected segments were flagged and independently re‐checked before export. The cleaned IBI series were then resampled at 4 Hz using cubic interpolation to generate an evenly spaced tachogram.

Frequency‐domain HRV was estimated in MATLAB using the Welch periodogram (pwelch function) with 100‐s Hamming windows, 50% overlap, and Hanning weighting, yielding ~0.01 Hz frequency resolution. Window length was chosen to match the duration of a shooting block, preserving low‐frequency components while minimizing non‐stationary drift. Each analysis window was fully contained within its respective block to prevent data spillover across conditions.

Spectral power was integrated within the low‐frequency (LF: 0.04–0.15 Hz) and high‐frequency (HF: 0.15–0.40 Hz) bands and expressed in both absolute units (ms^2^) and normalized units (n.u.). The LF/HF ratio was derived as a unitless index of sympathovagal balance. Time‐domain indices were also computed: the standard deviation of normal‐to‐normal intervals (SDNN, ms) as a measure of overall HRV reflecting both sympathetic and parasympathetic influences, and the root mean square of successive differences (RMSSD, ms) as a marker of short‐term, vagally mediated parasympathetic activity.

All HRV metrics were computed separately for each condition and averaged across trials. This analytic approach conforms to the standards of the Task Force of the European Society of Cardiology [31] and subsequent methodological reviews [32, 33], ensuring comparability with contemporary HRV research.

### 2.4 Performance Measures

Accuracy was defined as the proximity of each shot to the bullseye, quantified by the FATS system's 10‐point scoring algorithm. Precision was defined as radial error, calculated as the mean Euclidean distance of each shot from the participant's shot centroid within a block, indexing the degree of shot dispersion. Together, these measures captured outcome‐based proficiency, whereas gunpoint jerkiness indexed fine motor stability during the 4‐s pre‐shot aiming period.

### 2.5 Post‐Manipulation Check

The effectiveness of the motivational manipulation was verified through a brief post‐experiment survey. Participants ranked each condition (reward, punishment, neutral) on perceived stressfulness and enjoyment (1 = highest, 2 = moderate, 3 = lowest; no ties), confirming that the incentive contingencies were appraised as affectively distinct. This ranking served as a qualitative manipulation check, whereas the STAI‐S and HRV indices were analyzed separately as dependent measures of task‐induced affective and autonomic responses, reflecting broader psychophysiological regulation rather than immediate appraisal.

In addition, a brief post‐manipulation Likert‐scale survey further confirmed participants' subjective perception of reward and punishment framing. To statistically validate these perceptual distinctions, repeated‐measures ANOVAs with stress level and enjoyment level (high, medium, low) as within‐subject factors were conducted to examine their influence on behavioral and physiological outcomes.

### 2.6 Procedure

#### 2.6.1 Experimental Design

A within‐subjects design was employed to examine how motivational context (reward, punishment, neutral) modulated neural, physiological, and behavioral responses during a precision shooting task. Participants were tested individually in a controlled laboratory environment.

#### 2.6.2 Motivational Manipulation

**FIGURE 4:**
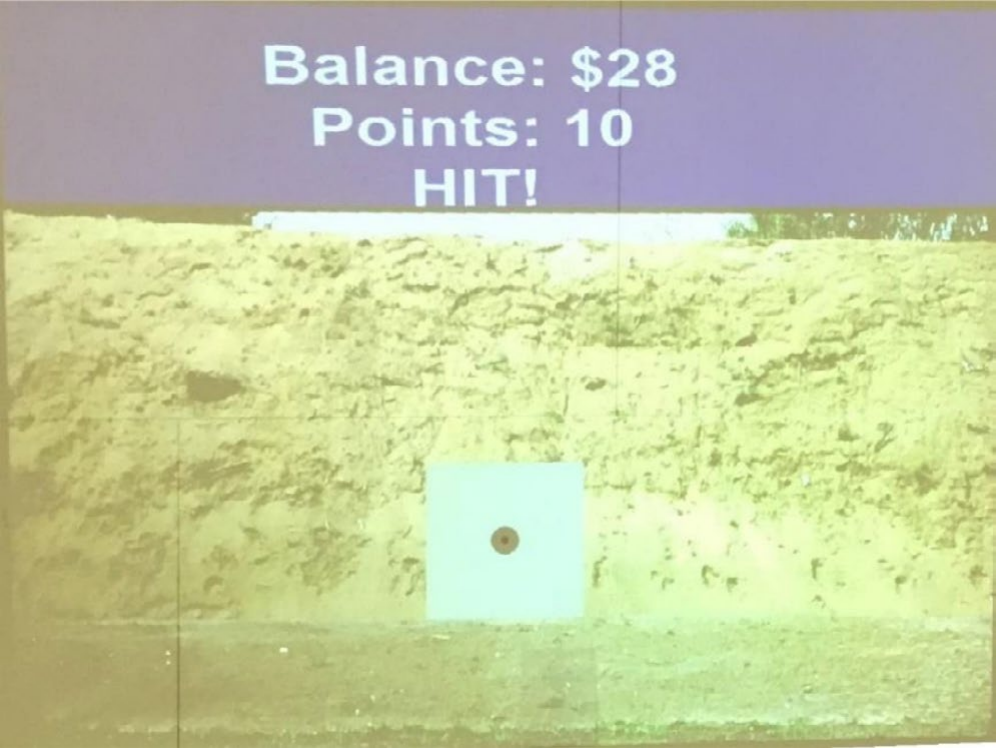
Image of shooting scenario and an example of feedback given in the reward condition.

**FIGURE 5:**
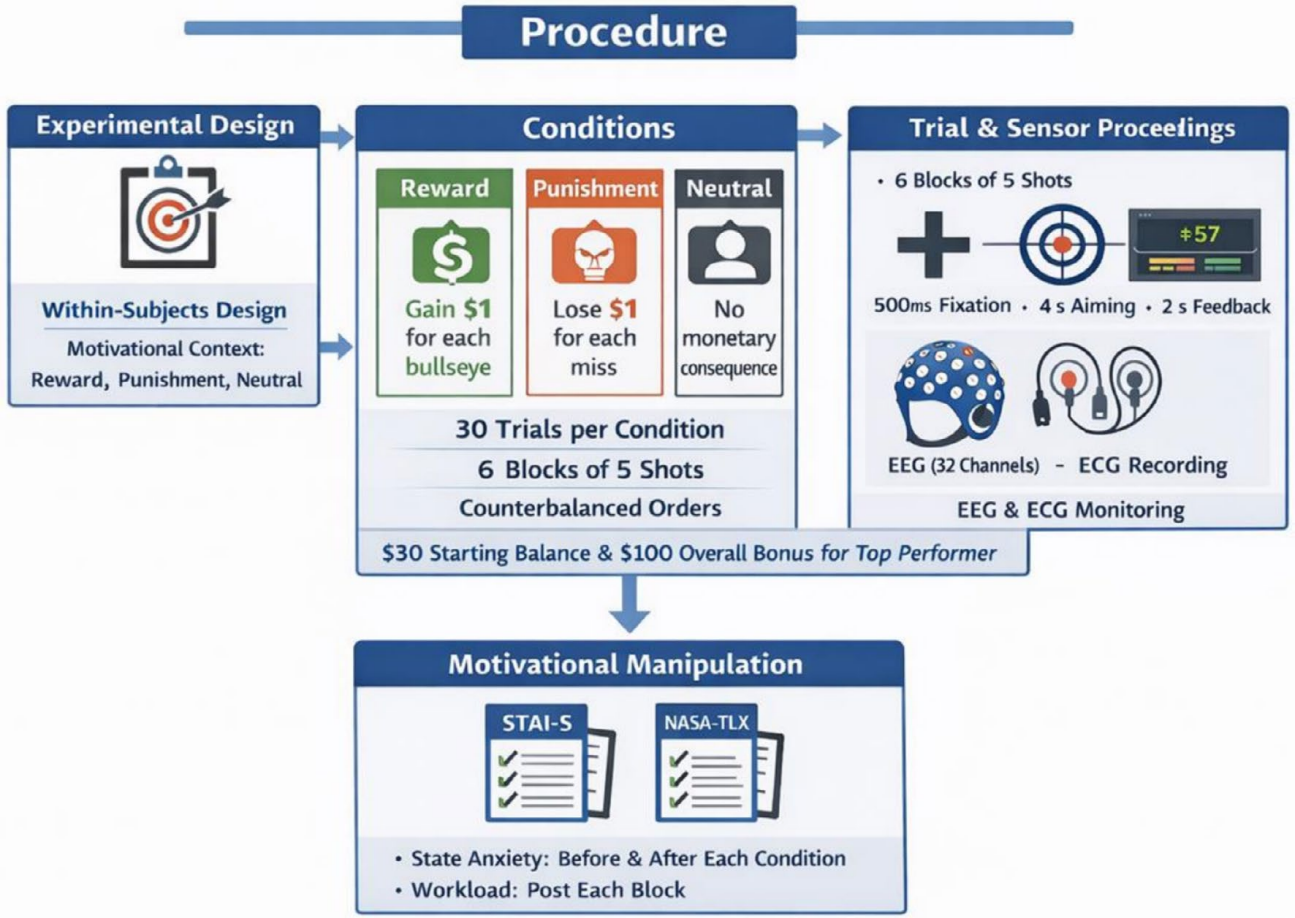
Flowchart summary of the experimental procedure.

Motivational context was operationalized using a performance‐based monetary incentive structure. Each participant completed 30 trials per condition, organized into six blocks of five shots. In the reward condition, participants gained $1 for each bullseye (10 points); in the punishment condition, they lost $1 for each shot outside the bullseye; and in the neutral condition, no monetary consequences were applied. All participants began with a $30 starting balance and were informed that the top overall performer would receive an additional $100 bonus (Figure 4). Condition order was fully counterbalanced using a 3 × 3 Latin square design, with participants randomly assigned to one of six possible presentation orders (Figure 5).

#### 2.6.3 Instructions and Practice

Standardized instructions were provided before each condition, outlining the performance contingencies. A brief practice block was administered to ensure comprehension of task rules and feedback displays.

#### 2.6.4 Trial Structure

Each trial began with a fixation cross (500 ms), followed by a 4‐s aiming period, trigger pull, and feedback presentation (1‐s delay, 1‐s duration). Feedback included hit/miss status, numerical score, current balance, and a trajectory display of the previous five trials. Condition‐specific reminders were presented midway through each block.

#### 2.6.5 Sensor Placement

EEG was recorded from a 32‐channel cap (10–20 system) covering frontal, central, temporal, and parietal regions. ECG was recorded using a three‐lead configuration (right clavicle, left lower rib, right lower rib). Electrode impedances were maintained below 10 kΩ.

#### 2.6.6 Psychological Assessments

State anxiety (STAI‐S) was assessed immediately before and after each condition, whereas perceived workload was measured using the NASA‐TLX following each block. As a manipulation check, participants completed a post‐experiment survey ranking the conditions in terms of perceived stressfulness and enjoyment, after which a structured debriefing was conducted.

#### 2.6.7 Ethical Approval

All procedures were approved by the University of Maryland Institutional Review Board, and written informed consent was obtained from all participants in accordance with the Declaration of Helsinki.

### 2.7 Data Analysis

#### 2.7.1 EEG Analysis

EEG data were segmented into five consecutive 1‐s epochs spanning from −4000 ms to +1000 ms relative to the trigger pull, consistent with temporal windows established in prior research [25]. The five epochs included: E4 (−4000 to −3000 ms), E3 (−3000 to −2000 ms), E2 (−2000 to −1000 ms), E1 (−1000 to 0 ms), and S1 (0 to +1000 ms), capturing both preparatory and immediate post‐movement neural dynamics.

Power spectral density was computed via Fast Fourier Transform (FFT) using 1‐s Hamming windows with 50% overlap and no zero‐padding, producing a frequency resolution of 1 Hz (i.e., contiguous spectral estimates at 1‐Hz intervals). A 10% Hanning taper was applied to minimize spectral leakage. Power values were log‐transformed (natural log) to normalize skewed distributions [34]. For each condition, spectra were baseline‐normalized to the eyes‐open resting period using a decibel ratio transform [10 × log_10_ (Power_task/Power_baseline)], rather than subtraction or percentage change, ensuring comparability across sessions.

Independent component analysis (ICA) was employed to correct ocular and muscle artifacts. Components were selected or rejected based on established criteria: those with frontal topographies and slow delta activity (eye blinks) or high‐frequency peripheral activity (muscle noise) were removed, whereas components reflecting neural activity (central/posterior alpha–theta topographies) were retained. Selection was guided by time‐course, scalp map, and spectral profile inspection, assisted by the ADJUST algorithm [35]. Trials with residual noise > ±100 μV were excluded (< 3% of total).

##### 2.7.1.1 FAA

FAA was computed as ln (F4 alpha power) − ln (F3 alpha power), indexing left–right frontal activation balance. FAA was calculated within each epoch and condition, then averaged across epochs and trials for group‐level comparisons.

##### 2.7.1.2 Temporal Alpha (T3 Alpha)

Alpha power (8–13 Hz) was extracted from electrode T3, located over the left temporal cortex, associated with verbal–analytic processing. To account for inter‐individual variability, each participant's individualized alpha frequency (IAF) was identified as the peak between 7.5 and 12.5 Hz [36]. Alpha at T3 was quantified as IAF ±1 Hz, providing subject‐specific precision.

##### 2.7.1.3 Sensorimotor Rhythm (SMR)

SMR (12–15 Hz) was extracted from Cz, overlying the sensorimotor cortex and indexing motor readiness/inhibition. Each participant's IAF was defined as the resting‐state alpha peak, and the SMR band was adjusted to IAF +2 to +5 Hz for individualized sensitivity to motor‐related activity.

##### 2.7.1.4 Frontal Midline Theta (Fm Theta)

Fm theta (4–7 Hz) was extracted from Fz, associated with cognitive control and attentional engagement. For individualized alignment, theta was defined as IAF −6 to IAF −3 Hz.

Power values for SMR and Fm theta were log‐transformed and expressed as relative power (proportion of total power) following baseline normalization.

##### 2.7.1.5 EEG Coherence

Functional connectivity was assessed via alpha‐band coherence between Fz–T3 and Pz–T3 within the individualized upper alpha band (IAF to IAF +2 Hz). Coherence was calculated in BrainVision Analyzer (Brain Products GmbH, Germany) using the normalized cross‐spectral density function with 1‐Hz frequency bins. Resulting coherence values were averaged across the specified frequency range per epoch and condition.

##### 2.7.1.6 Feedback‐Related Negativity (fERN)

Feedback‐locked ERPs were extracted at FCz from −500 to +1000 ms relative to performance feedback onset. Separate ERPs were averaged for HIT and MISS trials. fERN amplitude was computed as ERP (HIT) – ERP (MISS) within 250–350 ms post‐feedback, indexing feedback‐monitoring sensitivity.

#### 2.7.2 HRV Analysis

Heart rate variability (HRV) was quantified using standard time‐ and frequency‐domain indices in accordance with international guidelines [30, 32]. R‐peaks were identified using QRSTool v1.2.2 (Allen, University of Arizona) and visually verified, and any artifactual or ectopic beats deviating more than ±20% from the local mean were corrected using cubic‐spline interpolation. The cleaned inter‐beat interval (IBI) series was then resampled at 4 Hz in MATLAB R2022a (MathWorks, Natick, MA) to produce evenly spaced tachograms for spectral analysis. HRV was computed from contiguous 100‐s segments within each shooting block using 50%‐overlapped Hanning windows, yielding ~0.01‐Hz spectral resolution. Time‐domain metrics included the standard deviation of normal‐to‐normal intervals (SDNN), indexing overall variability, and the root mean square of successive differences (RMSSD), indexing vagally mediated parasympathetic activity. Frequency‐domain metrics were derived via Welch's periodogram (pwelch) and integrated over conventional bands, including low frequency (LF: 0.04–0.15 Hz) reflecting mixed sympathetic–parasympathetic modulation and high frequency (HF: 0.15–0.40 Hz) indexing parasympathetic activity, with the LF/HF ratio used to characterize sympathovagal balance. All HRV indices were averaged within each motivational condition and expressed in both absolute units (ms^2^) and normalized units following recommended reporting standards [33].

### 2.8 Data Quality and Reduction

All dependent variables (behavioral, EEG, HRV, EBS/EMG, NASA‐TLX, STAI‐S) were examined for normality (Shapiro–Wilk), variance homogeneity (Levene's test), and completeness prior to inferential analysis. Outliers exceeding ±3 SD were inspected; those due to transient noise were excluded listwise, whereas others were winsorized. Missing data were minimal (< 2%) and unsystematic.

After preprocessing and artifact correction, EEG, HRV, EBS/EMG, and behavioral measures were averaged within each condition for each participant. Log or z‐transformations were applied when normality violations persisted. Behavioral indices were standardized to comparable scales. This ensured that all physiological and performance measures were artifact‐free, normalized, and suitable for statistical testing.

### 2.9 Statistical Analysis

All statistical analyses were conducted using SPSS Version 26.0 (IBM Corp., Armonk, NY). The significance level was set at *α* = 0.05, and effect sizes are reported as partial eta squared (*ηp* ^2^).

Residuals from each model were tested for normality (Shapiro–Wilk) and homogeneity of variance (Levene's test). Greenhouse–Geisser corrections were applied when the assumption of sphericity (Mauchly's test) was violated. Observations exceeding ±3 SD from the cell mean were examined individually; those attributable to transient signal noise were excluded listwise (< 3% of total), and remaining extreme values were winsorized to the nearest valid observation. Missing data were minimal (< 2%) and not systematically distributed across conditions.

Because several dependent variables were analyzed within related conceptual domains (e.g., multiple shooting‐performance indices, HRV parameters, EEG metrics), each domain was treated as a separate family of outcomes. To control for familywise Type I error while preserving interpretability, Bonferroni‐adjusted post hoc tests were applied to significant effects, and Benjamini–Hochberg false‐discovery‐rate (FDR) corrections were used across related ANOVAs within each family.

Three‐level repeated‐measures ANOVAs were used for all within‐subject factors (Condition: reward, punishment, neutral) and included Performance Group (superior, inferior) as a between‐subject factor.

- *Behavioral and autonomic responses*: total shooting score, performance consistency, motor‐control precision, heart rate, SDNN, RMSSD, and LF/HF ratio.
- *Psychological measures*: STAI‐State anxiety (pre‐ and post‐condition) and NASA‐TLX workload subscales.
- *EEG indices*: FAA, T3 alpha power, SMR (Cz), frontal midline theta (Fz), alpha‐band coherence (Fz–T3, Pz–T3), and fERN (FCz). EEG analyses included Epoch (E4 to S1) as a within‐subject factor where applicable.

Significant main or interaction effects were followed by Bonferroni‐adjusted simple‐effects comparisons. For clarity, only effects that survived Bonferroni/FDR correction are reported as significant; others are described as *nonsignificant trends*.

A 2 (Outcome: Hit vs. Miss) × 3 (Condition: reward, punishment, neutral) repeated‐measures ANOVA examined neural differences between successful and unsuccessful shots for high‐alpha power (8–13 Hz) at T3 and alpha coherence (Fz–T3, Pz–T3).

Finally, Pearson correlations were computed to explore relationships among cortical indices (FAA, SMR, frontal theta, fERN, coherence), autonomic markers (HRV metrics), behavioral outcomes (total score, consistency, precision), and subjective states (workload, anxiety) across motivational contexts. Correlations were interpreted descriptively to illustrate functional coupling across neural, autonomic, and behavioral systems.

## 3 Results

### 3.1 Motivational Conditions Manipulation

Manipulation checks confirmed that motivational conditions (reward, punishment, neutral) effectively modulated participants' affective states, influencing motor performance. Lower stress levels were associated with more consecutive bullseye shots (*F*(2, 58) = 4.04, *p* < 0.05, *ηp* ^2^ = 0.12), whereas higher enjoyment levels correlated with greater accuracy (*F*(2, 58) = 4.64, *p* < 0.05, *ηp* ^2^ = 0.14) and consistency (*F*(2, 58) = 6.38, *p* < 0.01, *ηp* ^2^ = 0.18). These findings demonstrate that reduced stress and increased enjoyment enhance motor performance in a homogeneous expert cohort.

### 3.2 Shooting Performance

**FIGURE 6:**
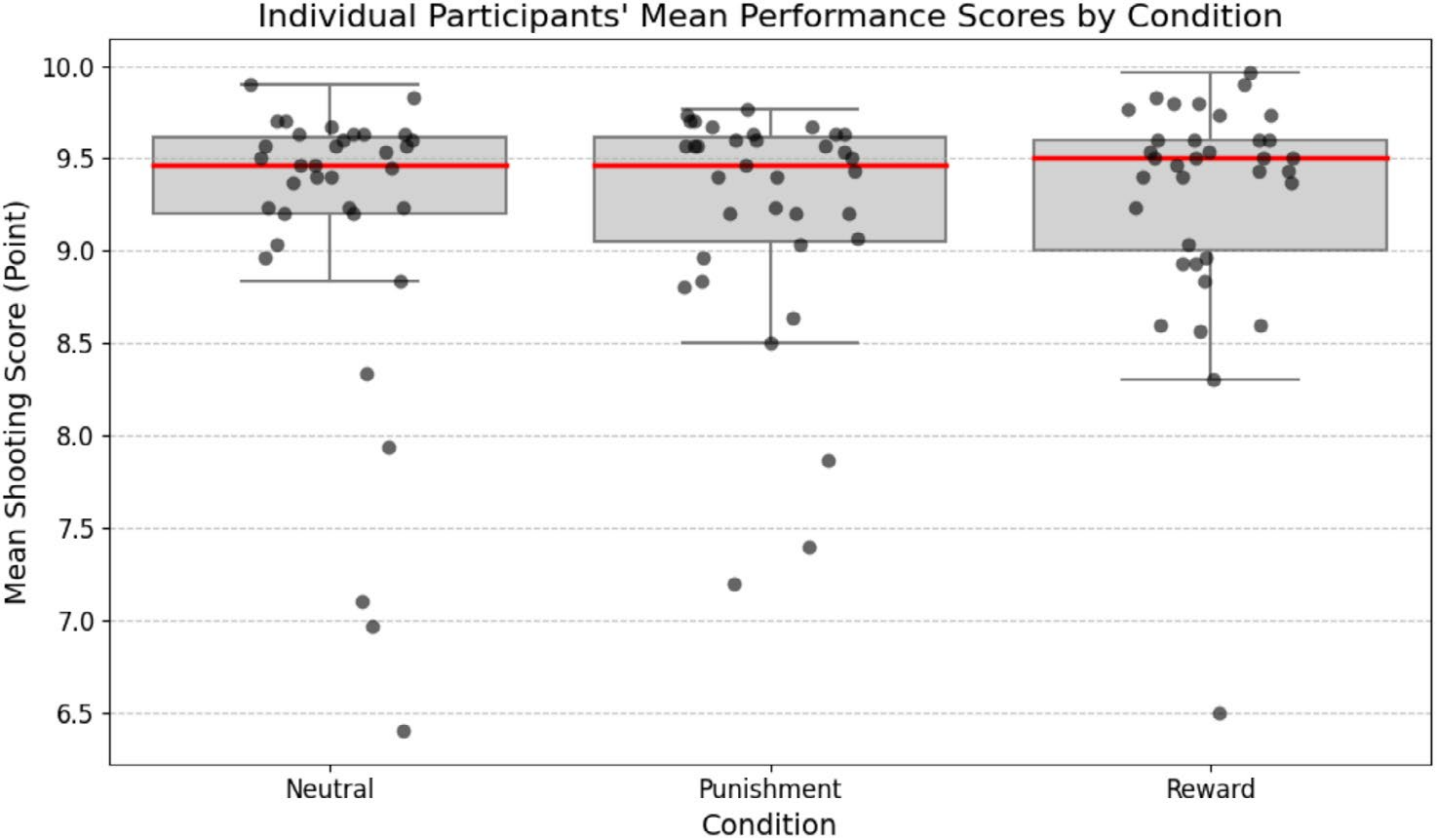
Individual participants' mean shooting scores by condition. Each black dot represents one participant's mean shooting score in that condition. The overlying box plot shows the distribution, and the horizontal red line marks the median score for all 35 participants in that condition.

A repeated‐measures ANOVA revealed that the Superior group significantly outperformed the Inferior group in total scores, bullseye hits, and consecutive bullseye sequences across reward, punishment, and neutral conditions (total scores: *F*(1, 30) = 32.38, *p* < 0.01, *ηp* ^2^ = 0.52; bullseyes: *F*(1, 30) = 59.07, *p* < 0.01, *ηp* ^2^ = 0.66; consecutive bullseyes: *F*(1, 30) = 19.33, *p* < 0.01, *ηp* ^2^ = 0.39). These results indicate greater accuracy and consistency in the Superior group (Figure 6). Group means and standard deviations are presented in Table 1.

**TABLE 1:** Mean shooting scores and standard deviations by group and condition.

Group	Condition	*M*	SD
Inferior	Neutral	9.38	0.38
Inferior	Punishment	9.26	0.41
Inferior	Reward	9.41	0.40
Superior	Neutral	9.37	0.45
Superior	Punishment	9.49	0.21
Superior	Reward	9.63	0.31

### 3.3 EEG and EMG Indices

#### 3.3.1 T3 Alpha Power (8–13 Hz)

A repeated‐measures ANOVA on T3 alpha power revealed a significant main effect of Epoch, *F*(4, 120) = 6.15, *p* < 0.001, *ηp* ^2^ = 0.170, indicating time‐dependent modulation around shot execution. Post hoc Bonferroni‐corrected comparisons showed peak alpha power at E1 (−1000 to 0 ms) compared to E4 (−4000 to −3000 ms; *p* < 0.01, *d* = 0.65) and reduced power at S1 (0 to +1000 ms) relative to E1 (*p* < 0.01, *d* = 0.70), reflecting enhanced sensorimotor readiness and reduced verbal–analytic interference during shot preparation.

A significant main effect of Condition, *F*(2, 60) = 4.25, *p* = 0.019, *ηp* ^2^ = 0.124, indicated higher T3 alpha power in the neutral condition compared to reward and punishment conditions (*p* < 0.05), suggesting greater verbal–analytic disengagement under motivational stress. The Performance Group main effect approached significance, *F*(1, 30) = 3.96, *p* = 0.055, *ηp* ^2^ = 0.117, with superior performers trending toward lower T3 alpha power, supporting the psychomotor efficiency hypothesis.

A significant Condition × Epoch interaction, *F*(8, 240) = 2.81, *p* = 0.006, *ηp* ^2^ = 0.086, revealed that T3 alpha dynamics varied by motivational context, with the reward condition showing the steepest pre‐shot alpha suppression, followed by punishment, and minimal suppression in the neutral condition. No significant Performance Group × Epoch interaction, *F*(4, 120) = 1.42, *p* = 0.228, *ηp* ^2^ = 0.045, or Condition × Group × Epoch interaction, *F*(8, 240) = 1.17, *p* = 0.317, *ηp* ^2^ = 0.038, was observed.

#### 3.3.2 SMR Power (12–15 Hz at Cz)

A repeated‐measures ANOVA on SMR power at Cz revealed a significant main effect of Epoch, *F*(4, 120) = 17.14, *p* < 0.001, *ηp* ^2^ = 0.434, with SMR power increasing progressively during the aiming period and peaking at E1 (−1000 to 0 ms). A significant main effect of Condition, *F*(2, 60) = 4.12, *p* = 0.021, *ηp* ^2^ = 0.121, showed higher SMR power in reward and punishment conditions compared to neutral. The Performance Group main effect was not significant, *F*(1, 30) = 3.41, *p* = 0.075, *ηp* ^2^ = 0.102. Significant Condition × Epoch (*F*(8, 240) = 2.98, *p* = 0.005, *ηp* ^2^ = 0.090) and Performance Group × Epoch (*F*(4, 120) = 12.50, *p* = 0.004, *ηp* ^2^ = 0.182) interactions indicated differential SMR modulation by motivational context and performance level.

Simple main effect analyses revealed that superior performers exhibited higher SMR power from E3 (−3000 to −2000 ms) through S1 (0 to +1000 ms), particularly in reward and punishment conditions, with significant group differences at E3 (*t*(33) = 2.25, *p* = 0.032, *d* = 0.80), E2 (*t*(33) = 2.76, *p* = 0.010, *d* = 0.90), E1 (*t*(33) = 2.45, *p* = 0.020, *d* = 0.87), and S1 (*t*(33) = 2.50, *p* = 0.018, *d* = 0.89). Conversely, inferior performers showed higher SMR power at E4 (−4000 to −3000 ms; *t*(10) = 3.51, *p* < 0.01, *d* = −1.24), suggesting less efficient early motor preparation.

#### 3.3.3 Frontal Midline Theta (4–7 Hz at Fz)

A repeated‐measures ANOVA on frontal midline theta power (IAF −6 to −3 Hz) at Fz revealed a significant main effect of Epoch, *F*(4, 120) = 6.80, *p* < 0.001, *ηp* ^2^ = 0.185, with theta power peaking at E1 (−1000 to 0 ms) across conditions, reflecting heightened preparatory attention. Significant main effects of Condition, *F*(2, 60) = 3.95, *p* = 0.024, *ηp* ^2^ = 0.116, and Performance Group, *F*(1, 30) = 4.32, *p* = 0.046, *ηp* ^2^ = 0.126, indicated greater theta engagement in reward and punishment conditions and stronger modulation in superior performers.

Significant Condition × Epoch (*F*(8, 240) = 2.75, *p* = 0.007, *ηp* ^2^ = 0.084) and Performance Group × Epoch (*F*(4, 120) = 2.89, *p* = 0.026, *ηp* ^2^ = 0.088) interactions showed that theta dynamics varied by motivational context and performance level. Superior performers exhibited a steeper pre‐shot theta increase, particularly in reward and punishment conditions, whereas inferior performers showed a flatter trajectory. These findings suggest that frontal midline theta reflects attentional processes sensitive to motivational context and individual performance capacity.

#### 3.3.4 T3–Pz Coherence

**FIGURE 7:**
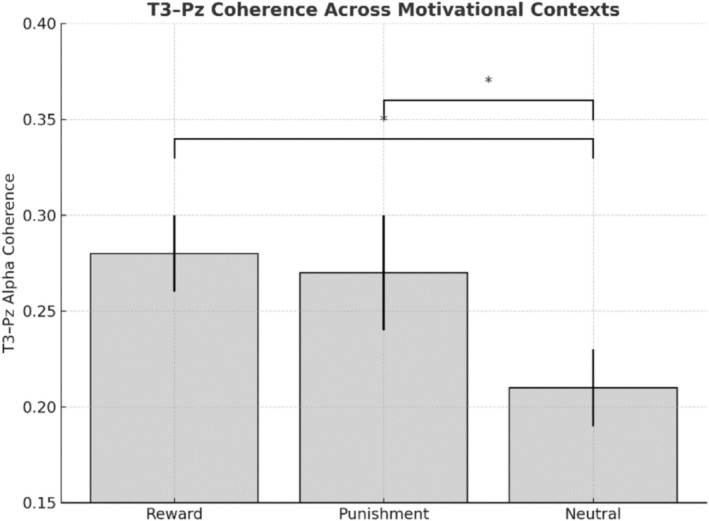
Comparison of T3–Pz coherence across Conditions. Error bars are 1 SD.

A repeated‐measures ANOVA on T3–Pz coherence in the individualized upper‐alpha band (IAF to IAF +2 Hz; within 8–13 Hz) revealed a significant main effect of Epoch, *F*(4, 120) = 6.08, *p* < 0.01, *ηp* ^2^ = 0.17. Coherence was lower at E1 (−1000 to 0 ms) relative to earlier epochs, indicating reduced parietal–temporal connectivity during shot preparation. A significant Condition × Epoch interaction, *F*(8, 240) = 4.06, *p* < 0.01, *ηp* ^2^ = 0.12, showed that coherence was lower in the neutral condition than in reward and punishment conditions. Post hoc comparisons confirmed that motivational context and task phase modulated parietal–temporal connectivity (see Figure 7).

#### 3.3.5 FAA

FAA (log F4 α–log F3 α) was computed as an index of relative left‐ versus right‐frontal activation. A 3 (Condition: reward, punishment, neutral) × 2 (Performance Group: superior, inferior) repeated‐measures ANOVA revealed no significant main or interaction effects (*p*s > 0.10), indicating that motivational context did not systematically alter frontal asymmetry across groups. This absence of modulation suggests that FAA may not be a sensitive marker of short‐term incentive framing in this task, despite prior evidence linking frontal asymmetry to approach–withdrawal motivation and affective–motor regulation in performance contexts [37, 38, 39].

#### 3.3.6 Feedback‐Related Negativity (fERN)

**FIGURE 8:**
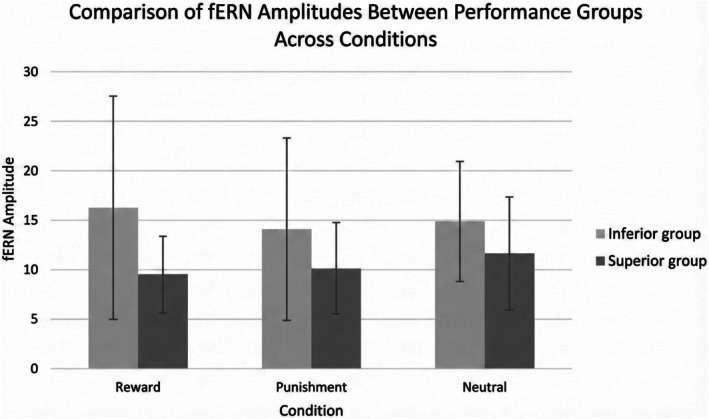
Comparison of fERN amplitudes between Performance Groups across Conditions. Error bars are 1 SD.

A significant main effect of Performance Group was found for fERN amplitude, *F*(1, 24) = 4.39, *p* < 0.05, *ηp* ^2^ = 0.15, with inferior performing participants exhibiting greater fERN amplitudes—indicative of heightened error sensitivity or evaluative reactivity (Figure 8).

### 3.4 Hit/Miss Comparisons

A repeated‐measures ANOVA on Fz–T3 alpha coherence (8–13 Hz) revealed a significant Hit/Miss × Performance Group interaction, *F*(1, 30) = 51.98, *p* < 0.01, *ηp* ^2^ = 0.63. Post hoc tests showed that superior performers had lower coherence during hits and higher coherence during misses, whereas inferior performers exhibited the opposite pattern (higher coherence during hits, lower during misses). Within hits, superior performers showed significantly lower coherence than inferior performers, *F*(1, 30) = 22.66, *p* < 0.01, *ηp* ^2^ = 0.43; within misses, superior performers had higher coherence, *F*(1, 30) = 7.60, *p* < 0.05, *ηp* ^2^ = 0.20.

For Pz–T3 alpha coherence, a significant main effect of Hit/Miss was found, *F*(1, 30) = 4.33, *p* < 0.05, *ηp* ^2^ = 0.13, with higher coherence during misses. A significant Hit/Miss × Performance Group interaction, *F*(1, 30) = 78.15, *p* < 0.01, *ηp* ^2^ = 0.72, mirrored the Fz–T3 pattern: superior performers showed lower coherence during hits (*F*(1, 30) = 24.80, *p* < 0.01, *ηp* ^2^ = 0.45) and higher coherence during misses (*F*(1, 30) = 13.23, *p* < 0.01, *ηp* ^2^ = 0.31) compared to inferior performers.

Analysis of behavioral indices revealed significant main effects of Hit/Miss on motor stability and performance consistency. Hits exhibited lower gunpoint jerkiness (*F*(1, 30) = 16.06, *p* < 0.01, *ηp* ^2^ = 0.35), higher precision (shorter shot‐to‐centroid distance; *F*(1, 30) = 30.00, *p* < 0.01, *ηp* ^2^ = 0.50), and greater accuracy (closer proximity to target center; *F*(1, 30) = 12.14, *p* < 0.01, *ηp* ^2^ = 0.29). Bonferroni‐corrected post hoc tests confirmed that hits had significantly reduced jerkiness, improved precision, and enhanced accuracy compared to misses, with medium‐to‐large effect sizes. No significant effects were observed for standard deviations of these measures.

### 3.5 Psychological and Subjective Workload

#### 3.5.1 STAI‐State Scores

A repeated‐measures ANOVA revealed a significant main effect of Condition on STAI‐State anxiety scores, *F*(3, 90) = 6.75, *p* < 0.01, *ηp* ^2^ = 0.18, with post hoc tests indicating higher anxiety in the punishment and neutral conditions relative to baseline. Anxiety was also significantly influenced by stress level, *F*(2, 58) = 7.12, *p* < 0.01, *ηp* ^2^ = 0.20, with lower anxiety reported under the least stressful condition. Similarly, Enjoyment had a significant effect on anxiety, *F*(2, 58) = 4.34, *p* < 0.05, *ηp* ^2^ = 0.13, with participants reporting lower STAI scores in the most enjoyable condition.

#### 3.5.2 Nasa‐TLX

Self‐reported workload analysis revealed a significant main effect of Performance Group on frustration ratings, *F*(1, 30) = 5.87, *p* < 0.05, *ηp* ^2^ = 0.16, with the inferior performing group reporting higher frustration (*M* = 54.58, SD = 4.45) than the superior performing group (*M* = 37.50, SD = 4.69). A significant main effect of stress level on frustration was also found, *F*(2, 58) = 3.64, *p* < 0.05, *ηp* ^2^ = 0.11; however, Bonferroni‐corrected post hoc comparisons did not identify significant pairwise differences. For self‐evaluated performance, stress level showed a significant main effect, *F*(2, 58) = 6.27, *p* < 0.01, *ηp* ^2^ = 0.18, with participants rating their performance as closer to failure in the most stressful condition (*M* = 39.42, SD = 2.80) compared to the medium (*M* = 30.67, SD = 2.60) and low‐stress conditions (*M* = 31.39, SD = 2.77).

#### 3.5.3 Physiological Arousal

A repeated‐measures ANOVA on mean heart rate revealed a significant main effect of Condition, *F*(2, 56) = 5.99, *p* < 0.01, *ηp* ^2^ = 0.18, with post hoc tests indicating higher heart rates in both the reward and punishment conditions compared to the neutral condition. These findings reflect increased physiological arousal under motivationally salient contexts.

### 3.6 Correlational Analysis

#### 3.6.1 Startle Reflex and Workload‐Related Associations

EBS amplitude consistently reflected affective and neurophysiological dynamics across motivational contexts. EBS was positively correlated with state anxiety in the punishment (*r*(29) = 0.50, *p* < 0.01, *r* ^2^ = 0.25), most stressful (*r*(29) = 0.51, *p* < 0.01, *r* ^2^ = 0.26), and least enjoyable (*r*(29) = 0.49, *p* < 0.01, *r* ^2^ = 0.24) conditions, indicating large effect sizes and underscoring its sensitivity to emotional stress. Additionally, EBS amplitude was negatively associated with Pz–T3 alpha coherence prior to shooting—at 1 s in the punishment condition (*r*(29) = −0.44, *p* < 0.05, *r* ^2^ = 0.19), and at both 3 and 1 s in the most stressful condition (*r*(29) = −0.55, *p* < 0.05, *r* ^2^ = 0.30; *r*(29) = −0.47, *p* < 0.05, *r* ^2^ = 0.22). A similar negative correlation emerged at 3 s in the least enjoyable condition (*r*(29) = −0.39, *p* < 0.05, *r* ^2^ = 0.15). These findings suggest that heightened affective reactivity coincides with reduced parieto‐temporal connectivity, indicative of cognitive inefficiency under threat. In the second most stressful condition, EBS was positively related to RMSSD (*r*(28) = 0.43, *p* < 0.05, *r* ^2^ = 0.18) and HF IBI variability (*r*(28) = 0.42, *p* < 0.05, *r* ^2^ = 0.18), potentially reflecting compensatory parasympathetic engagement.

#### 3.6.2 fERN and Workload‐Related Associations

fERN amplitude was associated with subjective workload across motivational contexts, supporting its role as a neural marker of perceived task difficulty and cognitive load. In the reward condition, fERN correlated with frustration (*r*(28) = 0.41, *p* < 0.05, *r* ^2^ = 0.17); in the punishment condition, with mental and temporal demands (*r*s = 0.37–0.38, *p*s < 0.05, *r* ^2^s = 0.14); and in the neutral condition, with physical demand (*r*(31) = 0.38, *p* < 0.05, *r* ^2^ = 0.14). Under the most stressful condition, fERN was associated with both mental and physical demands (*r*s = 0.36–0.39, *p*s < 0.05, *r* ^2^s = 0.13–0.15).

Temporal demand was further related to fERN in the second most stressful (*r*(28) = 0.43, *p* < 0.05, *r* ^2^ = 0.18) and most enjoyable (*r*(28) = 0.39, *p* < 0.05, *r* ^2^ = 0.15) conditions, whereas physical demand was correlated with fERN in the second most enjoyable (*r*(30) = 0.55, *p* < 0.01, *r* ^2^ = 0.30) and least enjoyable (*r*(30) = 0.39, *p* < 0.05, *r* ^2^ = 0.15) conditions. These findings indicate that fERN amplitude systematically tracks workload perception across contexts, with medium‐to‐large effect sizes reflecting stable relationships between neural feedback processing and subjective task demands.

#### 3.6.3 FAA, HRV, and Performance Stability

In the reward condition, FAA was positively correlated with RMSSD (*r*(30) = 0.57, *p* < 0.01, *r* ^2^ = 0.32), and a similar relationship was observed in the least stressful condition (*r*(29) = 0.46, *p* < 0.01, *r* ^2^ = 0.21). These findings suggest that greater left‐frontal cortical activation—reflective of approach motivation and affective stability—was associated with enhanced parasympathetic activity.

Additionally, HRV indices including SDNN and RMSSD, demonstrated significant positive correlations with shooting performance under both reward and most enjoyable conditions (Reward: SDNN, *r* = 0.52; RMSSD, *r* = 0.40; Enjoyable: SDNN, *r* = 0.57; RMSSD, *r* = 0.39; all *p*s < 0.05). In parallel, these measures showed negative correlations with performance variability (Reward: SDNN, *r* = −0.57; RMSSD, *r* = −0.43; Enjoyable: SDNN, *r* = −0.59; RMSSD, *r* = −0.40; all *p*s < 0.05), indicating that greater autonomic flexibility was linked to more consistent performance.

Moreover, HRV indices were positively correlated with T3 alpha power during pre‐shot epochs in the reward condition, suggesting that enhanced parasympathetic activity was associated with increased cortical disengagement from verbal–analytic processing—a neural signature of motor automaticity and attentional efficiency. Comparable associations were observed for both SDNN and RMSSD in the most enjoyable condition, further reinforcing the link between autonomic flexibility and cortical quieting under positive motivational states.

In contrast, under punishment and high‐stress conditions, the LF/HF ratio—indicative of sympathetic dominance—was positively associated with motor instability. Specifically, higher LF/HF ratios correlated with greater gunpoint jerkiness (mean: *r*(30) = 0.41, *p* < 0.05, *r* ^2^ = 0.17; SD: *r*(30) = 0.43, *p* < 0.05, *r* ^2^ = 0.18) and increased shot deviation from the target center (*r* range = 0.37–0.40, all *p*s < 0.05, *r* ^2^ = 0.14–0.16). These findings suggest that heightened sympathetic arousal impairs fine motor control and reduces movement consistency, especially under threat‐related motivational conditions. The observed medium to medium‐to‐large effect sizes further underscore the reliability of these associations.

## 4 Discussion

This study demonstrates that motivational context plays a pivotal role in shaping the neural, autonomic, and behavioral architecture of precision performance under stress. By integrating multiple neurophysiological markers—FAA, SMR, fERN, fronto‐temporal coherence, EBS, and HRV—the findings reveal how coordinated system‐level regulation underpins performance resilience. These results align with key propositions from the psychomotor efficiency hypothesis [8], the neurovisceral integration model [40], and the CAM framework [7]. Collectively, the evidence underscores that optimal performance in high‐pressure environments depends on the effective orchestration of affective regulation, cognitive control, and motor readiness.

The between‐group differences highlight that superior shooting performance extends beyond technical skill, reflecting advanced self‐regulatory capacities across affective, cognitive, and motor domains. Superior performers exhibited a neurophysiological profile marked by optimized attentional focus, emotional resilience, and motor efficiency. Specifically, they showed lower fERN amplitudes, indicating reduced maladaptive error sensitivity and greater adaptability to performance demands [41]. Elevated SMR power during the pre‐shot period further reflected enhanced motor readiness [42, 43], whereas reduced fronto‐temporal coherence suggested effective disengagement from verbal–analytic interference—hallmarks of psychomotor efficiency [16].

FAA emerged as a crucial index of affective regulation in this context. Reward framing consistently enhanced left‐frontal activation, supporting approach motivation and emotional stability. This pattern aligns with the prefrontal asymmetry literature, where leftward asymmetry is associated with adaptive engagement and resilience, buffering against the disruptive impact of stressors on performance [19]. The inverse association between FAA and fERN amplitude further substantiates the role of affective regulation in modulating error sensitivity, suggesting that top‐down cortical control may mitigate evaluative threat processing.

SMR modulation provided robust evidence of motor readiness and psychomotor efficiency, reinforcing its central role in stable and precise execution under stress [25, 44]. The temporal ramping of SMR power during preparation—especially pronounced in superior performers—reflects efficient cortical gating that suppresses task‐irrelevant motor pathways and stabilizes neuromuscular activity [9, 42]. This mechanism reduces sensorimotor noise, enabling smoother and more automatic motor output, a hallmark of expert performance across precision‐demanding domains [45].

Higher and more sustained SMR engagement in successful trials and among superior shooters suggests that SMR actively contributes to performance optimization by supporting refined motor control and attentional stability, rather than merely reflecting skill. This aligns with the psychomotor efficiency hypothesis, which posits that experts achieve superior outcomes through economical neural resource allocation, minimizing cognitive and emotional interference [8].

Moreover, SMR's sensitivity to motivational framing—showing greater engagement under reward—underscores its role in the integrated brain–body mechanisms through which incentives shape performance states. These findings position SMR as a promising and trainable target for interventions such as neurofeedback [46, 47, 48] and brain–computer interface training, which can enhance motor consistency, reduce variability, and accelerate skill acquisition [21, 44]. By directly influencing preparatory neural states, SMR‐based interventions hold translational potential for elite sport, military training, rehabilitation, and motor learning contexts.

The modulation of frontal midline (Fm) theta observed in this study underscores its critical role in cognitive control and attentional engagement during precision performance under motivational stress. Elevated theta power at Fz, ramping to a peak about 1 s before shot execution, reflects proactive recruitment of executive control for task‐focused attention and performance monitoring [49]. Generated primarily by the anterior cingulate and medial prefrontal regions, this preparatory activity supports conflict resolution, goal maintenance, and distraction suppression—functions essential for cognitive readiness and stable motor execution under pressure [11, 12, 50]. Superior performers showed consistently higher theta engagement, indicating more effective activation of control processes that stabilize performance through enhanced attentional regulation [51]. This pattern aligns with the psychomotor efficiency hypothesis, suggesting experts optimize cortical engagement to meet task demands without excessive interference [8]. Moreover, the sensitivity of Fm theta to motivational context—with greater modulation under reward and punishment than neutral conditions—supports the CAM model [7] and MAP model [52], highlighting how incentive salience recalibrates cognitive effort and resource allocation. Collectively, these findings position Fm theta as a key marker of adaptive preparation and performance resilience [53], reinforcing its potential as both a diagnostic index of expertise and a target for neurofeedback‐based focus training [54, 55, 56].

The fronto‐temporal coherence results highlight key neurocognitive mechanisms of cognitive interference and motor control under stress. Elevated alpha‐band coherence between the frontal midline (Fz) and left temporal (T3) regions—most evident in inferior performers and during punishment—reflects maladaptive verbal–analytic engagement that disrupts motor automaticity and smooth execution [16, 57]. This heightened coupling between executive and verbal–analytic systems signals conscious, effortful processing that interferes with the seamless integration of cognitive and motor functions [58]. Consistent with the CAM model, such stress‐induced shifts toward explicit control compromise automaticity and reduce psychomotor efficiency [7].

In contrast, superior performers showed consistently reduced Fz–T3 coherence, particularly during successful trials, underscoring the value of functional decoupling between prefrontal monitoring and verbal–analytic regions [59]. This decoupling likely minimizes disruptive self‐talk and over‐analysis, enabling motor programs to unfold with greater automaticity and stability [9, 21, 25]. Overall, these findings suggest that expert performance depends not only on motor skill proficiency but also on the ability to suppress unnecessary cortical cross‐talk, thereby maintaining an optimal balance between attentional control and motor readiness under motivational stress [60].

The modulation of feedback‐related negativity (fERN) provides important insight into cognitive control during performance adaptation under motivational stress. Larger fERN amplitudes observed under punishment and among inferior performers reflect heightened evaluative threat processing and increased sensitivity to negative feedback—an overactivation of the anterior cingulate cortex error‐monitoring system [41]. This exaggerated error signaling may amplify anxiety, disrupt attentional focus, and impair motor execution, consistent with Attentional Control Theory, which posits that anxiety‐driven hypervigilance toward errors undermines attentional efficiency [61].

In contrast, superior performers exhibited attenuated fERN responses, suggesting more adaptive modulation of performance monitoring. Their rapid yet efficient error detection limited excessive evaluative engagement, preserving attentional resources and supporting motor automaticity [62]. This balanced error‐processing profile aligns with the psychomotor efficiency hypothesis, indicating that experts minimize unnecessary cognitive load to maintain stability under pressure [8]. Collectively, these findings position fERN as a sensitive marker of individual differences in error reactivity and cognitive control.

Autonomic flexibility, indexed by HRV, played a pivotal role in supporting resilience under motivational stress. Increases in RMSSD and SDNN, together with lower LF/HF ratios in reward contexts and among superior performers, reflect greater parasympathetic dominance and autonomic flexibility, supporting emotional composure and stable motor control under pressure [63]. Conversely, the sympathetic dominance observed under punishment framing (reduced HRV and elevated LF/HF ratio) corresponds with diminished performance and heightened physiological stress. These autonomic findings integrate seamlessly with the neurovisceral integration model [40], underscoring the role of prefrontal‐autonomic coupling in performance regulation [64].

Together, these findings advance a systems‐level understanding of performance regulation, demonstrating that superior shooters achieve optimal outcomes by orchestrating a coherent psychophysiological state characterized by affective regulation (indexed by FAA and EBS), cognitive control (reflected in fERN and fronto‐temporal coherence), and motor readiness (captured by SMR and HRV). This multi‐dimensional coordination aligns with the central proposition of the CAM model, which posits that peak performance arises from the dynamic interplay across emotional, cognitive, and motor systems rather than isolated domain‐specific processes [7]. The observed alignment between cortical, subcortical, and autonomic mechanisms supports not only performance stability but also the capacity to flexibly adapt to varying motivational demands, such as reward or punishment contexts—an adaptive feature that distinguishes expert performers from those more vulnerable to stress‐induced disruption.

Importantly, the translational implications of these findings extend beyond theoretical refinement, offering practical avenues for performance enhancement in high‐stakes environments. Interventions such as neurofeedback protocols targeting FAA, SMR, or fronto‐temporal coherence, alongside HRV biofeedback strategies, hold promise for actively cultivating the psychophysiological conditions that underpin resilience and psychomotor efficiency [21]. Such approaches may benefit not only elite shooters but also professionals in precision‐demanding fields—athletes, soldiers, surgeons—where focus, emotional control, and motor stability are vital. By targeting modifiable neural and autonomic markers, this framework supports the development of evidence‐based neurofeedback training to enhance performance under pressure. This interpretation is also consistent with emerging evidence that function‐specific neurofeedback protocols [65], particularly those targeting Mu‐rhythm dynamics, can yield improvements in visuomotor performance when neural states are explicitly linked to movement goals [66].

### 4.1 Limitations

Several limitations should be acknowledged when interpreting these findings, alongside opportunities for future research. First, the exclusive use of male ROTC cadets, whereas offering high experimental control and ecological validity for modeling operational stress, constrains generalizability across gender, age groups, and individuals without formal military training. Because expertise development and stress reactivity may vary by demographic and experiential factors, future work should examine whether the observed neurophysiological patterns extend to broader populations including novices and athletes from diverse performance domains [67].

Second, although the Meggitt Firearms Training System (FATS) provided a controlled and standardized platform for assessing motivational framing, it cannot fully replicate the unpredictability, emotional salience, and environmental complexity of real‐world high‐pressure contexts. Caution is therefore warranted when extrapolating these results to live‐fire or dynamic operational scenarios. Moreover, the trial‐by‐trial feedback inherent to the FATS system may have introduced adaptive learning effects. Superior performers may have leveraged immediate feedback to fine‐tune execution, whereas inferior performers may have over‐compensated following errors, potentially reducing within‐condition consistency. Future studies should consider manipulating feedback timing (e.g., delayed, aggregated, or withheld feedback) or modeling trial‐level learning curves to disentangle motivational influences from feedback‐driven adjustments.

Third, the seated shooting design enhanced internal validity by minimizing postural confounds and isolating neural mechanisms, but it reduced ecological validity by excluding postural control, a critical component of real‐world expertise.

Fourth, the use of a 32‐channel EEG montage, whereas sufficient for capturing general cortical dynamics, limited spatial resolution and precluded precise source localization. Future research should employ high‐density EEG or multimodal neuroimaging approaches to refine spatial precision and clarify the contributions of prefrontal, cingulate, and limbic circuits implicated in psychomotor efficiency and neurovisceral integration models.

Finally, the correlational design precludes causal inference. Although the associations between neurophysiological markers and performance outcomes support predictions from the CAM model and psychomotor efficiency theory, longitudinal and interventional approaches are needed to establish causality. Future studies should move beyond correlational observations to investigate the causal dynamics among key neural and autonomic markers—particularly the interplay between FAA, SMR, and fERN in shaping attentional control, emotional regulation, and behavioral stability. Neurofeedback and biofeedback interventions targeting these markers may provide mechanistic insight into how modulating specific brain states facilitates adaptive performance under pressure [68, 69].

Taken together, addressing these limitations will advance a more comprehensive, system‐level understanding of how motivational context, neurocognitive control, and autonomic flexibility interact to support optimal performance. Such mechanistic insights are essential not only for refining theoretical frameworks such as the CAM model and neurovisceral integration model but also for informing the design of targeted interventions to enhance psychomotor efficiency, resilience, and adaptive functioning in high‐stakes environments.

## 5 Conclusion

In conclusion, this study provides novel mechanistic evidence that motivational framing—through reward and punishment contingencies—dynamically modulates the neurocognitive and autonomic processes that support performance under pressure. Consistent with the psychomotor efficiency hypothesis and the CAM model, reward contexts promote a coordinated profile of cortical efficiency, characterized by elevated SMR, adaptive FAA–HRV coupling, higher HRV, and reduced ERN and fronto‐temporal coherence. This integrated psychophysiological state reflects optimal attentional engagement, emotional regulation, and motor readiness—hallmarks of expert performance under demanding conditions.

Conversely, punishment contexts elicited defensive hyperarousal, characterized by sympathetic dominance, heightened error monitoring, and disrupted cortical synchronization—patterns particularly pronounced in lower‐performing individuals. This maladaptive response aligns with threat appraisal models and attentional control theory, highlighting how excessive evaluative vigilance and cognitive interference degrade automaticity and performance stability under stress.

Overall, these findings demonstrate that motivational incentives recalibrate brain–body dynamics across affective, cognitive, and motor systems. Markers such as FAA, SMR, fERN, coherence, and HRV emerge as actionable targets for neurofeedback and biofeedback interventions designed to enhance self‐regulation, resilience, and execution in high‐stakes environments such as elite sport, military operations, and surgical performance.

## 6 Perspectives

These findings advance current understanding of performance under pressure by demonstrating that motivational framing systematically recalibrates psychophysiological regulation across neural, autonomic, and motor systems. Consistent with prior EEG and autonomic research in precision sport performance, reward‐based contexts promoted a coordinated state marked by efficient motor preparation, attenuated error monitoring, and enhanced autonomic flexibility, whereas punishment induced defensive arousal and maladaptive cortical coupling. By integrating electrophysiological and cardiac markers, the present results extend challenge–threat and psychomotor efficiency frameworks, identifying conditions under which motivational intensity shifts from adaptive engagement to performance‐disruptive overstress. Importantly, the combined EEG–HRV signatures observed here provide actionable indicators of stress resilience, cognitive–motor efficiency, and performance readiness during repeated high‐demand exposure. Such markers hold translational relevance for optimizing training load, monitoring vulnerability to performance breakdown, and informing individualized neurofeedback and biofeedback interventions aimed at sustaining execution stability and long‐term adaptability in high‐stakes performance environments.

## Funding

This study was supported by the Graduate Research Initiative Project, Department of Kinesiology, University of Maryland.

## Conflicts of Interest

The authors declare no conflicts of interest.

## Data Availability

Data sharing not applicable to this article as no datasets were generated or analysed during the current study.
